# Has the COVID-19 Pandemic Affected the Prevalence of Diabetic Ketoacidosis in Polish Children with Newly Diagnosed Type 1 Diabetes? An Example of the Largest Polish Pediatric Diabetes Center (Upper Silesia—Katowice, Poland)

**DOI:** 10.3390/healthcare10020348

**Published:** 2022-02-11

**Authors:** Ewa Rusak, Sebastian Seget, Maksymilian Macherski, Natalia Furgał, Przemysław Dyś, Przemysława Jarosz-Chobot

**Affiliations:** Department of Children’s Diabetology, Medical University of Silesia, 40-752 Katowice, Poland; sebastian.seget@sum.edu.pl (S.S.); maksm123@gmail.com (M.M.); natalia.furgal@gmail.com (N.F.); przemekdys20@gmail.com (P.D.); przemka1@o2.pl (P.J.-C.)

**Keywords:** children, COVID-19, type 1 diabetes, diabetic ketoacidosis

## Abstract

Purpose: The aim of this study was to analyze the prevalence of diabetic ketoacidosis (DKA) in children with newly disclosed type 1 diabetes (T1D) during the COVID-19 pandemic in 2020 compared to 2019. Methods: A retrospective analysis of the history database of all hospitalized children in our department. The International Society for Pediatric and Adolescent Diabetes (ISPAD) guidelines were used for the diagnosis of DKA. Results: The database of children with newly disclosed T1D included 196 patients (89 girls and 107 boys) from 2019, and 223 patients (113 girls and 110 boys) from 2020 (a total of 419 patients—202 girls and 217 boys) aged 0 to 18 years. A significantly higher percentage of DKA was observed in 2020 compared to the previous year (47.53% vs. 35.2% [*p* = 0.005]). The percentage of severe DKA increased in 2020 compared to 2019 (18.39% vs. 14.07% [*p* = 0.118]). Compared to 2019, the average HbA1c level was higher in 2020 (12.57 ± 2.75% vs. 11.95 ± 2.89% [*p* < 0.025]), and the average pH level (7.26 vs. 7.31 [*p* = 0.002], and average HCO3 level (16.40 vs. 18.66 [*p* = 0.001]) were lower, respectively. Conclusions: During the COVID-19 (2020) pandemic, the incidence of DKA increased in Polish children with newly diagnosed T1D. The conclusions from the analysis of the functioning of health systems during the pandemic should be used in the future to prevent, in similar periods, an increase in severe complications of delayed diagnosis of T1D.

## 1. Introduction

One of the coronaviruses—SARS-CoV-2 (severe acute respiratory syndrome coronavirus 2) spread rapidly around the world, causing a worldwide pandemic of COVID-19 (coronavirus disease 2019), which was confirmed by the World Health Organization (WHO) on 11 March 2020. Nevertheless, children and adolescents, having a milder, often asymptomatic course of COVID-19, also have a lower risk of hospitalization than adults [1,2,3]. However, it has been reported that in some children, the course of COVID-19 may be severe, including cases of multi-organ failure [3,4,5].

Simultaneously, according to the estimates of the International Diabetes Federation (IDF), over 1.1 million children and adolescents worldwide have Type 1 Diabetes (T1D) [6]. However, some studies indicate that both children and adolescents with diabetes are not more susceptible to SARS-CoV-2 infection than their non-diabetic peers [7,8,9].

To reduce the number of cases, many countries, including Poland, implemented numerous restrictions, aimed at limiting SARS-CoV-2 transmission by restraining interpersonal contacts. As a result of those limitations and the fear of being infected in a hospital, access to healthcare professionals in medical facilities was reduced [9,10]. Additionally, many guardians delayed seeking emergency help for their children, due to the reduction of service for non-COVID-19 patients [8].

One of the acute and life-threatening complications of diabetes is diabetic ketoacidosis (DKA). DKA is caused by a deficiency of circulating insulin and an increased concentration of counter-regulatory hormones, such as catecholamines or cortisol [11,12]. Progressive metabolic disorders in the course of DKA require urgent medical intervention. DKA may often be the result of previously undiagnosed T1D. In Poland, the prevalence of DKA in children with recent T1D disclosure was estimated at 22–36% [13,14,15], and in our region of Upper Silesia, this value fluctuates in the range of 23–33% [16,17]. In recent years in Europe, this value was at the level of 33–44% [18,19,20,21,22].

The COVID-19 pandemic dynamically changed the picture of medical care around the world, as well as the diagnosis of T1D in Polish children.

The aim of this study was to analyze the prevalence of DKA in children with newly disclosed T1D during the COVID-19 pandemic in 2020 compared to 2019.

## 2. Materials and Methods

A retrospective analysis of the medical history database of all hospitalized children in the Department of Children’s Diabetology and Pediatrics of the Upper Silesian Child Health Centre in Katowice, Department of Children’s Diabetology of the Medical University of Silesia in Katowice, Poland with a new diagnosis of T1D was carried out in the periods of 1 January to 31 December 2020 and 1 January to 31 December 2019.

The Department of Children’s Diabetology of the Medical University of Silesia in Katowice is the largest regional diabetes center for children and adolescents in Poland. It also has an international SWEET (an international network for pediatric diabetes center) reference certificate [23]. All children with newly diagnosed diabetes in the region of Upper Silesia are hospitalized in the local unit. Upper Silesia is the region of Poland with the highest level of urbanization (76.5% of the population lives in cities), population density (364 people/km^2^), and which covers 11.7% of the Polish population [24].

Selected parameters were assessed from the database: age, sex, blood glucose value on admission, level of glycated hemoglobin (HbA1c), pH of venous blood, and bicarbonate concentration.

The results of swabs for the presence of SARS-CoV-2 infection were not included in the analysis due to heterogeneous sampling in patients in 2020.

The International Society for Pediatric and Adolescent Diabetes (ISPAD) guidelines from 2018 were used as biochemical criteria for the diagnosis of DKA, that is, hyperglycemia (blood glucose concentration >200 mg/dL [11 mmol/L]; venous blood pH < 7.3 or serum bicarbonate concentration <15 mmol/L; ketonaemia (ß-hydroxybutyrate concentration in blood ≥3 mmol/L); and ketonuria [12]. Venous blood pH < 7.1 or bicarbonate <5 mmol/L were used to define severe ketoacidosis [12].

Children who had pre-diabetes or diagnosed diabetes other than T1D (not confirmed by the presence of at least one antibody out of three which were tested against: GAD (Glutamic Acid Decarboxylase), IA2 (Islet Antigen 2), ZnT8 (Zinc-Transporter 8)), as well as those whose medical history contained too little information (a total of seven people from 2019–2020) were excluded from the analysis. Ultimately, the database included 196 patients (89 girls and 107 boys) from 2019 and 223 patients (113 girls and 110 boys) from 2020 (a total of 419 patients—202 girls and 217 boys) aged 0 to 18 years.

The work was approved by the Bioethical Committee of the Medical University of Silesia PCN/0022/KB1/81/21.

Statistical calculations were performed using the Statistica 13.3 program. For the total statistical summary, the level of significance was *p* < 0.05. In the search for answers to the research questions, the analysis of differences with Student’s *t*-tests and the results of the chi-square test of independence were used.

## 3. Results

Characteristics of children with newly diagnosed T1D in 2019 and 2020 are presented in Table 1 and Table 2. The overall number of hospitalized children, gender, and mean age of diagnosis did not differ significantly. On the other hand, among children with newly diagnosed T1D, a significantly higher percentage of DKA was observed in 2020 compared to the previous year (47.53% vs. 35.2% [*p* = 0.005]). Although the percentage of severe DKA increased in 2020 compared to 2019, it was not statistically significant (18.39% vs. 14.07% [*p* = 0.118]).

**Figure 1:**
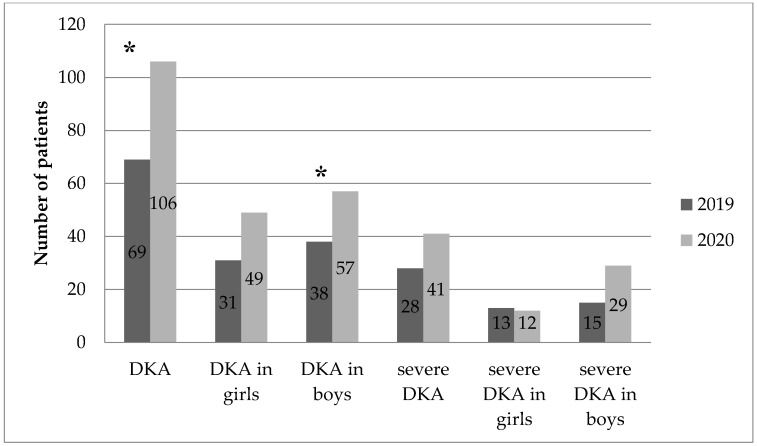
Number of DKA and severe DKA in children in 2019 and 2020. *—*p* < 0.05.

**Figure 2:**
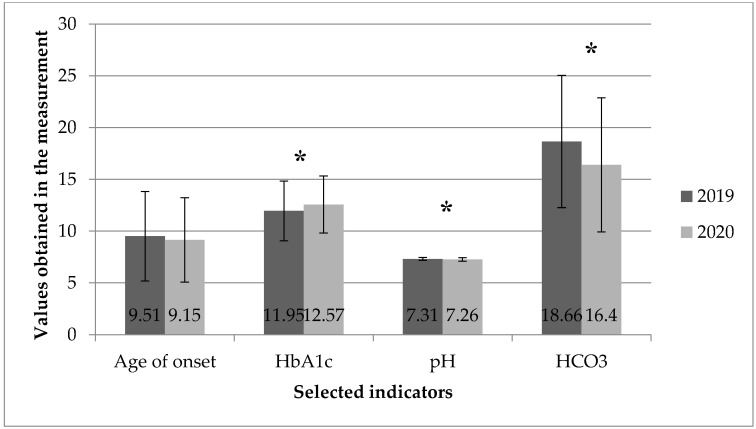
Mean values and standard deviation of the parameters of children at the time of clinical diagnosis of T1D: age, HbA1c, pH, HCO3 in 2019 and 2020. *—*p* < 0.05.

The average HbA1c level in patients with newly diagnosed T1D was significantly higher in 2020 than in 2019 (12.57 ± 2.75% vs. 11.95 ± 2.89% [*p* < 0.025]). In 2020, compared to the 2019 data, a significant decrease in the average pH level (7.26 vs. 7.31 [*p* = 0.002]) and HCO3 (16.40 vs. 18.66 [*p* = 0.001]) was also observed. The above results of the analyses are illustrated in Figure 1 and Figure 2.

**Figure 3:**
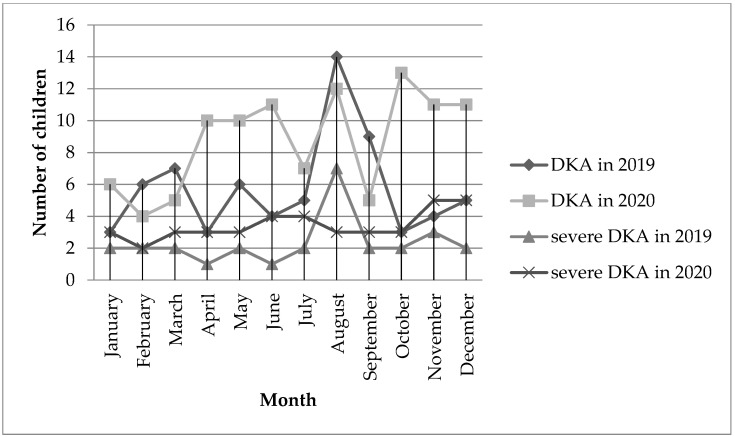
The number of DKA and severe DKA in children in 2019 and 2020 by month.

A comparative analysis specifying the number of patients with newly diagnosed T1D with DKA in individual months showed a significant increase in the percentage of DKA in children in October 2020 compared to the same month of the previous year (25% vs. 59% [*p* = 0.012]). The following months with a high increase in the percentage of patients with DKA in 2020 compared to 2019 are: April (27.2% vs. 66.7%) and November (20% vs. 64.7%). It should be noted that the periods with the greatest increase in the number of patients with DKA occur after the periods of strict restrictions in Poland (lockdown), that is, March and October. In order to visualize the occurrence of DKA and severe DKA in particular periods, the results are presented in Figure 3.

## 4. Discussion

This study is an attempt to assess the impact of the COVID-19 pandemic on the incidence of DKA in Polish children with newly diagnosed T1D. The Department of Children’s Diabetology in Katowice, being the largest regional pediatric diabetes center in Poland, with extensive, long-term experience and good cooperation in the region, represents Poland well. DKA is an acute, life-threatening condition that, if left untreated, leads to: severe water-electrolyte imbalance, hypovolemic shock, acute kidney failure, pulmonary edema, acute respiratory distress syndrome (ARDS), or cerebral edema [12,25,26]. The mortality rate in DKA oscillates between 0.15–0.3%, with the most common cause of death in 60–90% of brain edema [26,27,28]. One of the major risk factors for developing DKA is the delayed diagnosis of T1D [12]. In previous years in our region of Upper Silesia, DKA incidence was estimated at approximately 23–33% [16,17].

On 14 March 2020, the first total lockdown was introduced in Poland in order to reduce the spread of the SARS-CoV-2 virus. The reduced access to health care services, and delay in obtaining medical help by caregivers had a direct impact on the state of medical care. In March 2020, the number of new diagnoses of T1D at the Department of Children’s Diabetology of the Medical University of Silesia in Katowice was only 8, which most likely resulted in the fact that in the following month (April), the percentage of DKA increased to a dangerously high level compared to the previous year (an increase by 39.5 percentage points), and it was the highest in the year. DKA was present in almost 67% of children with newly diagnosed T1D. Year on year, the number of patients with DKA in relation to the total number of patients in a given year increased by almost 12.5 p.p. The number of cases of severe DKA also increased, although it did not reach statistical significance.

A similar increase in the number of DKA in children occurred in 2020 worldwide. For example, during the COVID-19 pandemic in Germany, there was a marked increase in the incidence of DKA in children with newly diagnosed T1D [29]. In the analyzed period, the frequency of DKA was 44.7% and was significantly higher compared to the previous two years (in 2019 it was 24.5%, while in 2018—24.1%). The incidence of severe DKA was also significantly higher than in the previous years, where in 2020, it was 19.4%; in 2019, 13.95%; and in 2018, 12.3% [29]. One of the retrospective cross-sectional studies conducted in Israel, which included 11 pediatric emergency departments, indicated that in the first few months of the pandemic (1 March to 31 May 2020), the number of DKA in children with newly disclosed and previously confirmed T1D increased compared to the same period in 2019 [30]. However, it should be emphasized that these data cover only three months of both 2019 and 2020, which undoubtedly makes it difficult to draw conclusions about later periods of the pandemic. The United Kingdom was another country where the prevalence of DKA in children during the first wave of COVID-19 was compared [31]. The patients included in the study were children under 18 years of age who were treated in one of the four compared hospitals in London. Admittedly, the data from this study included a total of only 47 children with newly diagnosed T1D—30 children before the pandemic and 17 children during the onset of the pandemic (23 March to 30 June 2020), but the number of DKA was significantly higher during the first wave of the pandemic in comparison to the period before it. Researchers at the Romanian reference center for children with T1D compared two groups of children newly diagnosed with T1D. The first group of children were diagnosed between March 2020 and February 2021, while the second were diagnosed between 2003 and 2019 [32]. As in most of the publications cited so far, the incidence of DKA in the Romanian study increased from 39.42% (in the pre-pandemic group) to 65.99% (in the pandemic group).

It is worth noting that some Polish researchers had similar observations and tendencies to ours. One study indicates that when comparing the periods March–May 2020 and the same period of the previous year, the incidence of DKA was higher by 12%, but the result was not statistically significant [33]. On the other hand, the incidence rate of T1D in one of the centers in southwest Poland in a few months of the COVID-19 pandemic (first four months of 2020) was comparable to previous years, while their clinical condition at the time of diagnosis was worse than in previous years. However, the analysis of a short period must be emphasized, and this may be why the results are different from ours [34].

A dangerous upward trend has also been observed in Australia, where the incidence of severe DKA increased from 5% (pre-pandemic) to 45% (pandemic) [35], while the overall incidence of DKA during the pandemic increased from 26% to 73%. However, a factor that should be taken into account when assessing data from Australia is the long distance that patients have to cover in order to reach medical centers in this country [35]. Moreover, the number of pediatric patients in these centers in the compared periods—from 1 March to 31 May—were small and amounted to, for example, 11 patients in 2020 or 9 patients in 2019. Therefore, such large differences in the prevalence of DKA on recognition may have resulted from a relatively small number of patients. In Saudi Arabia, a multicentre retrospective analysis found that the prevalence of DKA in children (2019 vs. 2020) increased by 10 p.p. from 73% to 83%, and the amount of heavy DKA increased from 13.3% to 26% [36]. It should be noted that the study was conducted in Saudi Arabia and concerned younger children, aged 1–14. The Canadian authors observed an increase in the incidence of DKA in patients under 18 years of age from 45.6% (2019) to 68.2% (2020) and, similarly to other countries, an increase in the rate of severe DKA cases was observed when comparing the same periods from 13.2% in 2019 to 27.1% in 2020 [37]. 

One study from Sweden, where a complete lockdown was not introduced, indicates that the percentage of children with HbA1c > 70 mmol/mol (>8.6%) did not increase in the first 7 months of 2020 [38]. Such findings may be the result of the policy applied in Sweden, which only recommended the isolation of people from the population most at risk of developing COVID-19. Similarly, in one study conducted in the USA (central Pennsylvania), the prevalence of DKA in the period before and during the pandemic was similar and amounted to 47% and 48%, respectively [39]. Perhaps such findings are the result of the variability of the population structure or the relatively small number of children (42 patients) from the pandemic group (1 March–14 September 2020). All studies discussed considered children with newly diagnosed T1D.

The average level of HbA1c in our study was higher in 2020 compared to 2019 in a statistically significant manner. The same observations were also seen in many other countries [31,32,35,36]. Venous blood pH was, in most of the cited publications, a criterion for the diagnosis of DKA. During the pandemic, this rate was significantly lower than in the pre-COVID-19 periods. Although this decrease was not statistically significant in every case, the trend towards lower pH during the pandemic was noticeable [30,31,32,35].

In the cited studies, it is mainly postulated that the fear of going to the hospital during the pandemic and the difficult access to medical services due to the limited activity of some facilities were the main reason for the increase in cases of delayed diagnosis of T1D. In Poland, for the same reasons, the period of strict restrictions caused by the coronavirus pandemic presumably contributed to the increase in the frequency of DKA from 35% in 2019 to 47.5% in 2020 and severe DKA from 14.3% to 17%. A statement from the Public Opinion Research Center study from July 2020 on medical care during a pandemic indicated that almost every seventh respondent (15%) had problems with making an appointment or obtaining the necessary medical advice due to the pandemic, and 37% of respondents declared that for the same reason, their treatment was cancelled or postponed [40]. Our results reveal the fact that even a very well-developed network of health care units (example of Upper Silesia, Poland) during the COVID-19 pandemic did not prevent a significant deterioration of diagnostic diabetes care.

Nonetheless, there are some limitations of our study. One of them is that we compared the entire years 2019 and 2020 with each other, but we therefore analyzed each month separately to detect any differences. Moreover, it was not possible to present the analysis in individual age groups due to their small size, which could have resulted in drawing unsupported conclusions.

## 5. Conclusions

During the COVID-19 (2020) pandemic, the incidence of DKA increased in Polish children with newly diagnosed T1D.

The conclusions from the analysis of the functioning of health systems during the pandemic should be used in the future to prevent, in similar periods, an increase in severe complications of delayed diagnosis of T1D. Additionally, further education of society (including caregivers of children) about the symptoms of hyperglycemia is necessary in order to be able to effectively prevent the development of DKA in the future.

## Figures and Tables

**Table 1:** Characteristics of children and the prevalence of diabetic ketoacidosis in 2019 and 2020 with an independence assessment by a chi-square test.

Variable	Year 2019		Year 2020		*p*
	N	%	N	%	
Number of girls	89	45.41	113	50.67	0.091
Number of boys	107	54.59	110	49.33	0.840
Total number of children	196	100	223	100	0.187
Number of girls with DKA	31	15.82	49	21.97	0.248
Number of boys with DKA	38	19.39	57	25.56	**0.016**
Total number of children with DKA	69	35.20	106	47.53	**0.005**
Number of girls with severe DKA	13	6.63	12	5.38	0.721
Number of boys with severe DKA	15	7.65	29	13.00	0.108
Total number of children with severe DKA	28	14.07	41	18.39	0.118

Annotation. The percentages values represent the percentage of observations in the entire group of a given year. N—number of observations, *p*—significance level, bolded values *p* < 0.05.

**Table 2:** Analysis of differences using Student’s t-test for data of independent measurements from 2019 and 2020 for selected indicators in children.

Testing Variable	Year 2019			Year 2020			*p*
	N	M	SD	N	M	SD	
Age of onset	196	9.51	4.32	222	9.15	4.08	0.372
HBA1c	189	11.95	2.89	221	12.57	2.75	**0.025**
pH	180	7.31	0.14	222	7.26	0.17	**0.002**
HCO_3_	175	18.66	6.39	203	16.40	6.48	**0.001**

Annotation. N—number of observations, M—mean, SD—standard deviation, *p*—significance level, bolded *p* < 0.05.

## Data Availability

Data are available from the corresponding author, upon reasonable request.

## References

[1] Lu X., Zhang L., Du H., Zhang J., Li Y.Y., Qu J., Zhang W., Wang Y., Bao S., Li Y. (2020). SARS-CoV-2 Infection in Children. N. Engl. J. Med..

[2] Ludvigsson J.F. (2020). Systematic Review of COVID-19 in Children Shows Milder Cases and a Better Prognosis than Adults. Acta Paediatr..

[3] Borrelli M., Corcione A., Castellano F., Fiori Nastro F., Santamaria F. (2021). Coronavirus Disease 2019 in Children. Front. Pediatr..

[4] Jutzeler C.R., Bourguignon L., Weis C.V., Tong B., Wong C., Rieck B., Pargger H., Tschudin-Sutter S., Egli A., Borgwardt K. (2020). Comorbidities, Clinical Signs and Symptoms, Laboratory Findings, Imaging Features, Treatment Strategies, and Outcomes in Adult and Pediatric Patients with COVID-19: A Systematic Review and Meta-Analysis. Travel Med. Infect. Dis..

[5] Hoang A., Chorath K., Moreira A., Evans M., Burmeister-Morton F., Burmeister F., Naqvi R., Petershack M., Moreira A. (2020). COVID-19 in 7780 Pediatric Patients: A Systematic Review. EClinicalMedicine.

[6] Williams R., Colagiuri S. (2019). International Diabetes Federation IDF Diabetes Atlas.

[7] Cardona-Hernandez R., Cherubini V., Iafusco D., Schiaffini R., Luo X., Maahs D.M. (2021). Children and Youth with Diabetes Are Not at Increased Risk for Hospitalization Due to COVID-19. Pediatr. Diabetes.

[8] Elbarbary N.S., dos Santos T.J., de Beaufort C., Agwu J.C., Calliari L.E., Scaramuzza A.E. (2020). COVID-19 Outbreak and Pediatric Diabetes: Perceptions of Health Care Professionals Worldwide. Pediatr. Diabetes.

[9] d’Annunzio G., Maffeis C., Cherubini V., Rabbone I., Scaramuzza A., Schiaffini R., Minuto N., Piccolo G., Maghnie M. (2020). Caring for Children and Adolescents with Type 1 Diabetes Mellitus: Italian Society for Pediatric Endocrinology and Diabetology (ISPED) Statements during COVID-19 Pandemia. Diabetes Res. Clin. Pract..

[10] Cherubini V., Gohil A., Addala A., Zanfardino A., Iafusco D., Hannon T., Maahs D.M. (2020). Unintended Consequences of Coronavirus Disease-2019: Remember General Pediatrics. J. Pediatr..

[11] Wolfsdorf J.I., Allgrove J., Craig M.E., Edge J., Glaser N., Jain V., Lee W.W., Mungai L.N., Rosenbloom A.L., Sperling M.A. (2014). Diabetic Ketoacidosis and Hyperglycemic Hyperosmolar State. Pediatr. Diabetes.

[12] Wolfsdorf J.I., Glaser N., Agus M., Fritsch M., Hanas R., Rewers A., Sperling M.A., Codner E. (2018). ISPAD Clinical Practice Consensus Guidelines 2018: Diabetic Ketoacidosis and the Hyperglycemic Hyperosmolar State. Pediatr. Diabetes.

[13] Niechciał E., Skowrońska B., Michalak M., Fichna P. (2019). Ketoacidosis at Diagnosis of Type 1 Diabetes in Children and Adolescents from Wielkopolska Province in Poland: Prevalence, Risk Factors and Clinical Presentation. Clin. Diabetol..

[14] Szypowska A., Dżygało K., Wysocka-Mincewicz M., Mazur A., Lisowicz L., Ben-Skowronek I., Sieniawska J., Klonowska B., Charemska D., Nawrotek J. (2017). High Incidence of Diabetic Ketoacidosis at Diagnosis of Type 1 Diabetes among Polish Children Aged 10-12 and under 5 Years of Age: A Multicenter Study. Pediatr. Diabetes.

[15] Wojcik M., Sudacka M., Wasyl B., Ciechanowska M., Nazim J., Stelmach M., Starzyk J.B. (2015). Incidence of Type 1 Diabetes Mellitus during 26 Years of Observation and Prevalence of Diabetic Ketoacidosis in the Later Years. Eur. J. Pediatr..

[16] Olak-Białoń B., Deja G., Jarosz-Chobot P., Buczkowska E.O. (2007). The occurrence and analysis of chosen risk factors of DKA among children with new onset of DMT1. Pediatr. Endocrinol. Diabetes Metab..

[17] Chumięcki M., Prokopowicz Z., Deja R., Jarosz-Chobot P. (2013). Frequency and clinical manifestation of diabetic ketoacidosis in children with newly diagnosed type 1 diabetes. Pediatr. Endocrinol. Diabetes Metab..

[18] Manuwald U., Schoffer O., Hegewald J., Große J., Kugler J., Kapellen T.M., Kiess W., Rothe U. (2019). Ketoacidosis at Onset of Type 1 Diabetes in Children up to 14 Years of Age and the Changes over a Period of 18 Years in Saxony, Eastern-Germany: A Population Based Register Study. PLoS ONE.

[19] Ješić M.D., Ješić M.M., Stanisavljević D., Zdravković V., Bojić V., Vranješ M., Trifunović D., Necić S., Sajić S. (2013). Ketoacidosis at Presentation of Type 1 Diabetes Mellitus in Children: A Retrospective 20-Year Experience from a Tertiary Care Hospital in Serbia. Eur. J. Pediatr..

[20] Choleau C., Maitre J., Filipovic Pierucci A., Elie C., Barat P., Bertrand A.-M., de Kerdanet M., Letallec C., Levy-Marchal C., Nicolino M. (2014). Ketoacidosis at Diagnosis of Type 1 Diabetes in French Children and Adolescents. Diabetes Metab..

[21] Schober E., Rami B., Waldhoer T. (2010). Diabetic Ketoacidosis at Diagnosis in Austrian Children in 1989–2008: A Population-Based Analysis. Diabetologia.

[22] Vukovic R., Jesic M.D., Vorgucin I., Stankovic S., Folic N., Milenkovic T., Sajic S., Katanic D., Zivic S., Markovic S. (2018). First Report on the Nationwide Incidence of Type 1 Diabetes and Ketoacidosis at Onset in Children in Serbia: A Multicenter Study. Eur. J. Pediatr..

[23] SWEET An International Network for Pediatric Diabetes Centers List of Members by Category—SWEET Certified Centers of Reference. https://www.sweet-project.org/members-list.php.

[24] Fryc J., Kalisz P., Przybyła M. (2021). Population Vital Statistics and Migrations in Śląskie Voivodship in 2020.

[25] Wolfgram P., MacDonald M. (2015). Severe Hypertriglyceridemia Causing Acute Pancreatitis in a Child with New Onset Type I Diabetes Mellitus Presenting in Ketoacidosis. J. Pediatr. Intensive Care.

[26] Gasler N., Barnett P., McCaslin I., Nelson D., Trainor J., Louie J., Kaufman F., Quayle K., Roback M., Malley R. (2001). Risk Factors for Cerebral Edema in Children with Diabetic Ketoacidosis. N. Engl. J. Med..

[27] Edge J.A. (2001). The Risk and Outcome of Cerebral Oedema Developing during Diabetic Ketoacidosis. Arch. Dis. Child..

[28] Edge J.A., Ford-Adams M.E., Dunger D.B. (1999). Causes of Death in Children with Insulin Dependent Diabetes 1990–96. Arch. Dis. Child..

[29] Kamrath C., Mönkemöller K., Biester T., Rohrer T.R., Warncke K., Hammersen J., Holl R.W. (2020). Ketoacidosis in Children and Adolescents with Newly Diagnosed Type 1 Diabetes During the COVID-19 Pandemic in Germany. JAMA.

[30] Jacob R., Weiser G., Krupik D., Takagi D., Peled S., Pines N., Hashavya S., Gur-Soferman H., Gamsu S., Kaplan O. (2021). Diabetic Ketoacidosis at Emergency Department Presentation During the First Months of the SARS-CoV-2 Pandemic in Israel: A Multicenter Cross-Sectional Study. Diabetes.

[31] McGlacken-Byrne S.M., Drew S.E.V., Turner K., Peters C., Amin R. (2021). The SARS-CoV-2 Pandemic Is Associated with Increased Severity of Presentation of Childhood Onset Type 1 Diabetes Mellitus: A Multi-Centre Study of the First COVID-19 Wave. Diabet Med..

[32] Boboc A.A., Novac C.N., Ilie M.T., Ieșanu M.I., Galoș F., Bălgrădean M., Berghea E.C., Ionescu M.D. (2021). The Impact of SARS-CoV-2 Pandemic on the New Cases of T1DM in Children. A Single-Centre Cohort Study. JPM.

[33] Dżygało K., Nowaczyk J., Szwilling A., Kowalska A. (2020). Increased Frequency of Severe Diabetic Ketoacidosis at Type 1 Diabetes Onset among Children during COVID-19 Pandemic Lockdown: An Observational Cohort Study. pedm.

[34] Zubkiewicz-Kucharska A., Seifert M., Stępkowski M., Noczyńska A. (2021). Diagnosis of Type 1 Diabetes during the SARS-CoV-2 Pandemic: Does Lockdown Affect the Incidence and Clinical Status of Patients?. Adv. Clin. Exp. Med..

[35] Lawrence C., Seckold R., Smart C., King B.R., Howley P., Feltrin R., Smith T.A., Roy R., Lopez P. (2021). Increased Paediatric Presentations of Severe Diabetic Ketoacidosis in an Australian Tertiary Centre during the COVID-19 Pandemic. Diabet. Med..

[36] Alaqeel A., Aljuraibah F., Alsuhaibani M., Huneif M., Alsaheel A., Dubayee M.A., Alsaedi A., Bakkar A., Alnahari A., Taha A. (2021). The Impact of COVID-19 Pandemic Lockdown on the Incidence of New-Onset Type 1 Diabetes and Ketoacidosis Among Saudi Children. Front. Endocrinol..

[37] Ho J., Rosolowsky E., Pacaud D., Huang C., Lemay J., Brockman N., Rath M., Doulla M. (2021). Diabetic Ketoacidosis at Type 1 Diabetes Diagnosis in Children during the COVID-19 Pandemic. Pediatr. Diabetes.

[38] Ludvigsson J. (2020). Effect of COVID-19 Pandemic on Treatment of Type 1 Diabetes in Children. Acta Paediatr..

[39] Bogale K.T., Urban V., Schaefer E., Bangalore Krishna K. (2021). The Impact of COVID-19 Pandemic on Prevalence of Diabetic Ketoacidosis at Diagnosis of Type 1 Diabetes: A Single-Centre Study in Central Pennsylvania. Endocrinol. Diabetes Metab..

[40] Research Report by the Public Opinion Research Center No. 88/2020—Healthcare during the Epidemic 2020. https://www.cbos.pl/PL/publikacje/public_opinion.php.

